# Genomic Selection for Growth Traits in Pacific Oyster (*Crassostrea gigas*): Potential of Low-Density Marker Panels for Breeding Value Prediction

**DOI:** 10.3389/fgene.2018.00391

**Published:** 2018-09-19

**Authors:** Alejandro P. Gutierrez, Oswald Matika, Tim P. Bean, Ross D. Houston

**Affiliations:** ^1^The Roslin Institute and Royal (Dick) School of Veterinary Studies, The University of Edinburgh, Edinburgh, United Kingdom; ^2^Weymouth Laboratory, Centre for Environment, Fisheries and Aquaculture Science (CEFAS), Weymouth, United Kingdom

**Keywords:** genomic selection, Pacific oyster, growth, GBLUP, SNP array

## Abstract

Pacific oysters are a key aquaculture species globally, and genetic improvement via selective breeding is a major target. Genomic selection has the potential to expedite genetic gain for key target traits of a breeding program, but has not yet been evaluated in oyster. The recent development of SNP arrays for Pacific oyster (*Crassostrea gigas*) raises the opportunity to test genomic selection strategies for polygenic traits. In this study, a population of 820 oysters (comprising 23 full-sibling families) were genotyped using a medium density SNP array (23 K informative SNPs), and the genetic architecture of growth-related traits [shell height (SH), shell length (SL), and wet weight (WW)] was evaluated. Heritability was estimated to be moderate for the three traits (0.26 ± 0.06 for SH, 0.23 ± 0.06 for SL and 0.35 ± 0.05 for WW), and results of a GWAS indicated that the underlying genetic architecture was polygenic. Genomic prediction approaches were used to estimate breeding values for growth, and compared to pedigree based approaches. The accuracy of the genomic prediction models (GBLUP) outperformed the traditional pedigree approach (PBLUP) by ∼25% for SL and WW, and ∼30% for SH. Further, reduction in SNP marker density had little impact on prediction accuracy, even when density was reduced to a few hundred SNPs. These results suggest that the use of genomic selection in oyster breeding could offer benefits for the selection of breeding candidates to improve complex economic traits at relatively modest cost.

## Introduction

Pacific oyster (*Crassostrea gigas*) is the most cultivated oyster species worldwide and has been introduced to many countries for aquaculture production (Troost, 2010). Global production of this species reached ∼0.6 M tones in 2016 (FAO, 2018). Given its importance, several selective breeding programs based on family and mass selection have been conducted for the improvement of economically important traits such as body weight, growth rate, survival and yield (Langdon et al., 2003; Evans and Langdon, 2006; Li et al., 2011; de Melo et al., 2016), showing an improvement of the target traits after 1–5 generations. As with other aquaculture species, the recent development of genomic tools opens up the possibility for incorporating genetic markers into breeding programs via genomic selection, resulting in improved selection accuracy and genetic gain (Goddard and Hayes, 2007).

In recent years, substantial effort has been put toward the development of genomic resources for Pacific oyster, which include a reference genome assembly (Zhang et al., 2012), genetic marker databases including microsatellites (Li et al., 2003; Sekino et al., 2003) and SNPs (Sauvage et al., 2007; Fleury et al., 2009; Wang J. et al., 2015) and low density linkage maps, containing both microsatellites and SNPs (Hubert and Hedgecock, 2004; Hedgecock et al., 2015). The recent development of two oyster SNP arrays; a combined-species medium density array for Pacific oyster and European flat oyster (*O. edulis)* (Gutierrez et al., 2017) and a high density array for Pacific oyster (Qi et al., 2017); raises the opportunity of rapidly collecting genotype data for many 1000s of SNP markers dispersed throughout the genome. Moreover, a high density linkage map containing ∼20 K SNPs has recently been created and aligned with the physical reference genome assembly (Gutierrez et al., 2018).

A limited number of quantitative trait locus (QTL) mapping studies have been performed to examine the genetic basis of growth related traits in Pacific oyster (Hedgecock et al., 2007; Guo et al., 2012; Wang and Li, 2017), generally indicating that these traits are polygenic in nature. With the recently developed genotyping resources, GWAS might have the potential to address some of the drawbacks that QTL mapping (based on linkage) has, particularly related to marker density. Moreover, GWAS are based on a population level linkage disequilibrium between markers and QTL, which could potentially facilitate the application of marker-assisted selection (MAS) in breeding programs. However, MAS based on polygenic traits is likely to be ineffective due to capturing only a small proportion of the genetic variation in the trait. Therefore, genomic selection may be a promising avenue for incorporating markers into shellfish breeding. In genomic selection, genome-wide SNP markers are used to generate a genomic relationship matrix which is utilized to predict genomic estimated breeding values (GEBVs) for individuals without phenotypes, based on training of the genomic prediction equation in a reference population with both phenotypes and genotypes (Goddard and Hayes, 2007). Given that genomic selection can be used to accurately predict breeding values even in the absence of trait or pedigree information, it may have high potential for oysters where routine pedigree recording can be difficult for two reasons. The first is that maintaining physically separate families is logistically difficult and expensive, and tagging of juvenile oysters is challenging. The second is that genotyping errors for traditional marker assays such as microsatellites are common, and several examples of incorrect pedigree assignment have been described, thought to be due to the high frequency of null alleles, ranging from 16 to 51% (Launey and Hedgecock, 2001; Hedgecock et al., 2004; Reece et al., 2004). Encouraging, the use of SNPs from the oyster SNP array platform was shown to be successful in the parental assignment in a limited number of Pacific oyster families (Gutierrez et al., 2018). The advantages of genomic selection over traditional pedigree-based approaches in terms of accuracy of the predictions observed for both livestock and aquaculture species for polygenic traits have been described for several aquaculture species (Ødegård et al., 2014; Tsai et al., 2015; Dou et al., 2016; Palaiokostas et al., 2016; Correa et al., 2017; Vallejo et al., 2017a). However, despite the importance of Pacific oyster to global aquaculture, no studies have yet evaluated the potential of genomic selection for breeding value prediction in this species.

The primary aim of the current study was to evaluate the potential use of genomic prediction in a population of Pacific oysters derived from a commercial hatchery. Several growth-related traits were evaluated as exemplar polygenic traits, including shell length (SL), shell height (SH), and wet weight (WW). The impact of SNP marker density on genomic prediction accuracy was evaluated, alongside strategies for selecting low density panels for potential improvement of genotyping cost-efficiency.

## Materials and Methods

### Source of Oysters

The population used in this study were derived from crosses between broodstock from a commercial oyster hatchery (Guernsey Sea Farms, United Kingdom) and were a subset of the samples used for analysis of resistance to *Ostreid Herpesvirus*, as described in Gutierrez et al. (2018). There were two sets of oyster crosses used in the study. The first comprised three pair crosses that were created at Cefas (from 3 sires and 2 dams) and then reared in separate tanks. Larvae were held in 5 L tanks with daily water renewal, daily feeding and constant aeration. Post settling (at roughly 2 weeks) these were moved to 10 L tanks with a constant flow of water and feed. Spat were handled every 2–3 weeks, when the tanks were cleaned. Feed was provided at the rate recommend in the manual for hatchery culture of bivalve mollusc, according to the spat density and water volume of each tank. Larvae were fed a mix of *Chaetoceros*, T-Isochrysis and Pavlova algae. The remaining crosses (from 14 sires and 14 dams) were obtained as spat from a mass spawning at Guernsey Sea Farms (GSF). Prior to settling, larvae were held in upwelling 2 L bottle system, with aeration and constant supply of feed according to standard hatchery procedure. Post settling, oysters were held at GSF for 3 weeks then delivered overnight to Cefas where they were held on mesh upwelling system in a large 60 L tank, with a constant flow of water.

For both groups of oysters reared at Cefas (from larval stage for the first group, from post-settlement stage for the second group), spat were handled every 2–3 weeks, when the tanks were cleaned. The oysters were drip fed a constant supply of mixed food including Pavlova, T-Isochrysis and Tetraselmis. Throughout the experiment, all sea water was filtered, UV treated, mixed to a salinity of 25 ppt with RO water, and aerated prior to use. Feed was provided at the rate recommend in the manual for hatchery culture of bivalve mollusc, according to the density of each tank. On several occasions, where algal stocks were low, food was supplemented with shellfish diet 1,800^1^. Measurements were taken at approximately 6 months. The differences in early life environment may have affected growth rate and were therefore accounted for in the statistical model described below. Parental assignment was performed as described in Gutierrez et al. (2018), resulting in the identification of 23 different full-sibling families in the population. All animals were reared in accordance with the United Kingdom Home Office regulations regarding the use of animals in experiments. The trial was carried out at the Centre for Environment, Fisheries and Aquaculture Science (Cefas, United Kingdom).

### Phenotypic Measurements

Shell measurements (SL and SH) were taken following a standard protocol for the measurement of oyster shells (Galtsoff, 1964). Wet weight (WW) was recorded during the DNA extraction procedures where the whole animal (excluding shell) was used.

### SNP Array Genotyping

Genome-wide SNP data were generated using the recently developed Affymetrix SNP array for oysters (Gutierrez et al., 2017), as described in Gutierrez et al. (2018). Briefly, genomic DNA was extracted from the whole oyster (minus the shell) using the RealPure genomic DNA extraction kit (Valencia, Spain), quantified on a Qubit fluorometer (Invitrogen) and the DNA integrity was checked on a 1% agarose gel. Array genotyping was carried out at Edinburgh Genomics, and quality control was performed using the Axiom Analysis Suite v2.0.0.35, following the “best practices workflow” with a sample and SNP call threshold of 90%. These settings resulted in 23,388 SNPs classified as good quality and therefore retained for downstream analyses. Post-filtering, the final dataset comprised 820 individuals with genotype and phenotype data.

### Genetic Parameter Estimation

Genetic parameters for the resistance traits were estimated using a linear mixed model approach fitting animal as a random effect using ASReml 4 (Gilmour et al., 2015) with the following model:

(1) y=Xb+Zu+e

where **y** is the observed trait, **u** is the vector of additive genetic effects, **b** is the vector of fixed effect of tank, **e** is the residual error, and **X** and **Z** the corresponding incidence matrices for fixed effects and additive genetic effects, respectively. The (co)variance structure for the genetic effect was calculated either using the pedigree matrix (**A**) (i.e., **u**∼N(0, Aσ_a_^2^) or genomic matrix (G) N(0, Gσ_*a*_^2^), where σ^2^ is the genetic variance. Hence, the narrow sense heritability was estimated by the additive genetic variance and total phenotypic variance, as follows:

(2) $$h^{2}=\sigma^{2}{}_{a}/\sigma^{2}{}_{P}$$

where σ^2^_a_ is the additive genetic variance and σ^2^_p_ is the total phenotypic variance which is a sum of σ_*a*_^2^ + σ_*e*_^2^. The genomic relationship matrix used for the analysis was obtained according to VanRaden (2008) using the GenABEL package (Aulchenko et al., 2007) and inverted using a standard “R” function. The fixed effect of “tank” partially accounted for the differences in early life conditions between the pair-cross and batch-spawned oysters, which were held in separate tanks at Cefas during post-settlement rearing.

### Genome-Wide Association Studies

The GWAS were performed for the three growth-related traits using two approaches, first using the GenABEL package (Aulchenko et al., 2007) in R and also genomic BLUP analysis implemented in BLUPF90 software (Misztal et al., 2002). The genotype data was filtered as part of quality control by using the *check.markers* module to exclude SNPs with a minor allele frequency (MAF) <0.05, call rate <0.90 and significantly deviated from Hardy–Weinberg Equilibrium <1 × 10^−6^, leaving 13,278 SNPs for downstream analyses. Association analyses were performed using the family-based score test for association (FASTA) using the mmscore function (Chen and Abecasis, 2007) with the mixed linear model (MLM) approach used to avoid potential false positive associations due to population structure. Genotype data were used to calculate the genomic kinship matrix which was fitted in the model alongside the top four principal components as covariates to account for population structure. The genome-wide significance threshold was set to 3.76 × 10^−6^ as determined by Bonferroni correction (0.05/N), where N represents the number of QC-filtered SNPs across the genome, while the suggestive threshold was set as 3.76 × 10^−5^ (0.5/N), i.e., allowing 0.5 false positive per genome scan. For the BLUPF90 approach, the same data previously filtered by GenABEL were used. Model (1) was fitted using the genomic (G) relationship matrix that was created according to VanRaden (2008). In this case, windows of 10 adjacent (not overlapping) SNPs based on the linkage map position were created using POSTGSF90 (Aguilar et al., 2014). It has been shown that the use of a higher number of SNPs (as SNP windows) in the analysis of quantitative traits could capture the QTL effect more accurately than a single SNP (Habier et al., 2011). Even though recent studies have argue that the use of a higher number of markers in the window should provide better power for the detection of QTL (Wang H. et al., 2014; Gonzalez-Pena et al., 2016; Vallejo et al., 2017b). We chose to only use 10 consecutive SNPs (as windows) to reduce the possibilities of wrong SNP position, given that numerous assembly errors have been described within the oyster genome (Hedgecock et al., 2015; Gutierrez et al., 2018), and therefore the SNP order may not be fully accurate.

### Genomic Prediction

For the estimation of genomic prediction values, the genotype data was filtered to allow markers with a (MAF) >0.01, which resulted in a higher number of markers in the analysis (16,079 SNPs). Estimated breeding values were obtained using either pedigree-based BLUP (PBLUP) or Genomic best linear unbiased prediction (GBLUP) using the linear model described above. The accuracy of genomic selection was estimated by fivefold cross validation (training set 80%, validation set 20%), which were repeated five times. Phenotypes from the validation population were masked and breeding values were estimated using ASReml 4 using the linear mixed model described above (1). Prediction accuracy was calculated as the correlation between the predicted EBVs of the validation set and the actual phenotypes divided by the square root of the heritability estimated in the validation population [∼r(y1, y2)/h]. Mean prediction accuracy values obtained from the different sets were computed and compared between the pedigree and genomic approaches.

Two different strategies for evaluating the potential of lower marker densities for genomic prediction were applied. First, the low density SNP panel for use in the computing the genomic relationship matrix was selected by a progressive increase of the MAF threshold from 0.01 to 0.475 resulting in a progressive reduction in number of markers (as shown in **Table 2**); Secondly, the low density SNP panel was selected using a strategy of random “thinning” of SNPs from the full dataset (15, 10, 5, 2.5, 1, and down to 100 SNPs).

### Data Availability

Genotype data corresponding to these samples has already been made publicly available as **Supplementary Material** by Gutierrez et al. (2018). The combined-species medium density SNP array for oysters can be ordered from Thermo Fisher Scientific (Waltham, CA, United States).

## Results and Discussion

### Trait Summary and Heritability

The mean and standard deviation values for the growth-related traits were 11.27 ± 2.9, 34.94 ± 28.33, and 8.3 ± 1.97 for SH, WW, and SL, respectively. The genetic correlation between the traits was high >0.9 for all traits and the phenotypic correlation slightly lower (**Table 1**). Moderate heritability values were estimated for all traits, ranging from 0.20 (pedigree-based) for length, to 0.35 for weight (G-matrix based), as shown in **Table 1**. Previous studies have described significant heritability values for growth traits in Pacific oyster, albeit these estimates range in magnitude. For instance, Dégremont et al. (2007) reported the heritability estimates for weight that ranged from 0.07 to 0.15 in 6–8 months old *C. gigas*, while Kong et al. (2015) and Lannan (1972) reported values for weight at ∼0.35 in older samples of the same species. Other studies have reported heritability estimates for weight ranging from 0.1 to 0.5 in *C. gigas* (Hedgecock et al., 1991; Sheridan, 1997; Langdon et al., 2003; Evans and Langdon, 2006). Our results are consistent with heritability estimates described for shell measurements, e.g., Li et al. (2011) reported values of 0.149–0.402 for SH in *C. gigas* at 12 months of age, while Kong et al. (2015) described estimates of 0.49 for SH and 0.36 for SL, and Xu et al. (2017) reported heritability values of 0.18 for SH and 0.25 for SL. These results imply that growth can potentially be improved by selective breeding. Most oyster breeding programs are focused on increasing flesh weight, but since a high genetic correlation between the three analyzed traits (>0.90) was observed, selection (either pedigree or genomic based) for any one of the traits is likely to co-select for improvement of the other traits. High genetic and phenotypic correlations between these traits have also been observed previously (Kong et al., 2015), and (combinations of) these traits can be used to predict soft-body WW which is a main breeding objective for oysters (Wang X. et al., 2014).

**Table 1:** Genetic parameter estimates for the growth-related traits in the Pacific oyster samples.

	SH	SL	WW
Mean (s.d)	11.27 (2.9)	8.3 (1.9)	34.94 (28.3)
**HERITABILITY^b^**			
G-matrix	0.26 (0.05)	0.23 (0.06)	0.35 (0.05)
A-Matrix	0.23 (0.12)	0.20 (0.11)	0.31 (0.13)
**CORRELATION^a^**			
SH	–	0.83 (0.01)	0.78 (0.02)
SL	0.95 (0.04)	–	0.74 (0.02)
WW	0.92 (0.06)	0.90 (0.06)	–

*^a^Genetic correlation was estimated based on the genomic relationship matrix and values are shown below the diagonal, while phenotypic correlation values are shown above the diagonal. ^b^Heritability was estimated based on the genomic relationship matrix (G-matrix) and the pedigree.*

It should be noted that the oysters used to assess early-life growth in the current were potentially influenced by differences in early life environment, and by the fact that they were survivors of an OsHV-1 challenge experiment (Gutierrez et al., 2018). However, there was no evidence of genetic correlation between the traits of survival and the growth-related traits (data not shown), which is consistent with the findings of Dégremont et al. (2007). The early life conditions of the batch-spawned oysters and the laboratory spawned oysters were different, and this was partially accounted for by the inclusion of the fixed effect of “tank” in the statistical model (since the batch-spawned and pair-cross oysters were held in separate tanks at Cefas post-settlement). While this fixed effect was not significant, these early life differences may have impacted on the results, since genotype by environment interaction has been shown to be important for growth traits in Pacific oyster (Langdon et al., 2003; Dégremont et al., 2007). In addition, the disease challenge experiment may have had a minor influence on size, since growth during challenge may not be the same trait as growth in the absence of disease challenge. While the traits measured may not be a reliable indicator of oyster growth under commercially relevant conditions, they were heritable and polygenic and therefore served purpose for testing genomic prediction approaches.

### Genome-Wide Association Studies (GWAS)

There was no significant or suggestive association detected by GenABEL between any SNP and any of the three analyzed traits (**Supplementary Table S1** and **Supplementary Figure S1**). Additionally, the proportion of variance explained (PVE) by each SNP was low (∼2%) highlighting the absence of any major QTL controlling growth traits in this population. Moreover, BLUPF90 analysis based on consecutive (not overlapping) SNP windows did not detect major QTL in any of the three traits, although a suggestive QTL was detected for SL in a window that explained 1.48% of the genetic variance and located on LG 1 (48.61 cM) (**Supplementary Table S2** and **Supplementary Figure S2**), Worth noting that 2 windows located at LG 7 (73.23 cM) and LG 6 (39.94–40.82 cM) were found among the 10 highest scores for the three analyzed traits. These results suggest a polygenic nature of growth-related traits in Pacific oyster, controlled by many loci with small effects as it has been observed in many aquaculture species such as Atlantic salmon (Gutierrez et al., 2015; Tsai et al., 2015; Yoshida et al., 2017), rainbow trout (Wringe et al., 2010), common carp (Palaiokostas et al., 2018), grouper (Yu et al., 2018), turbot (Sánchez-Molano et al., 2011), Asian seabass (Wang L. et al., 2015), and also oysters (Guo et al., 2012; Li and He, 2014).

To inform a strategy for deployment of genetic markers in commercial aquaculture breeding programs, it is important to first define the genetic architecture of the target trait(s) in question. Marker assisted selection (MAS) is most appropriate when major QTL explain a high proportion of genetic variation in the trait, enabling use of a small panel of QTL-linked markers to supplement pedigree-based selection. A good example of this is resistance to the Infectious Pancreatic Necrosis virus (IPNV) in Atlantic salmon, where most genetic variation is explained by a single QTL (Houston et al., 2008; Moen et al., 2009). However, most growth-related traits (and other traits of economic importance to aquaculture production) are polygenic, and therefore genomic selection incorporating all markers to predict breeding values is likely to be a more effective approach.

### Genomic Prediction

**FIGURE 1:**
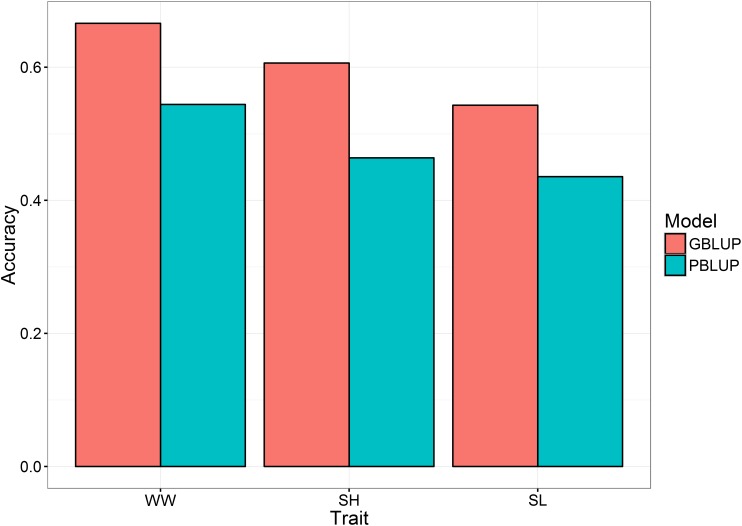
Mean accuracies for GBLUP and PBLUP for the three analyzed traits. WW, wet weight; SH, shell height; SL, shell length.

Genomic prediction accuracy for the three traits were tested using genotype information from 16,076 markers that passed the QC filter (MAF >0.01, CallRate >0.9). Animals were randomly split into training (80%) and validation (20%) sets for cross-validation, and this was repeated five times. The genomic prediction accuracy results highlighted that the higher prediction accuracies were obtained using the genomic information (G-matrix) than using the pedigree information (A-matrix) (**Figure 1**), with an increase of ∼25% for WW (from 0.54 PBLUP to 0.67 GBLUP) and SL (from 0.44 PBLUP to 0.54 GBLUP) to ∼30% for SH (from 0.47 PBLUP to 0.60 GBLUP). These results are in agreement with published literature which shows the increase in breeding value prediction accuracy using genomic vs. pedigree prediction in aquaculture species, e.g., Atlantic salmon (Ødegård et al., 2014; Yoshida et al., 2017; Barría et al., 2018; Robledo et al., 2018), rainbow trout (Vallejo et al., 2017a; Yoshida et al., 2018), sea bream (Palaiokostas et al., 2016). For growth traits in particular, studies in Atlantic salmon (Tsai et al., 2015), common carp (Palaiokostas et al., 2018), and Pacific white shrimp (Wang et al., 2017) have reported an increase in the prediction accuracies by the use of genomic information. The results of the current study highlight the potential of genomic selection for economic traits in oysters, albeit the cost may be prohibitively expensive and more cost-effective genotyping strategies may be required for feasibility of commercial application.

**FIGURE 2:**
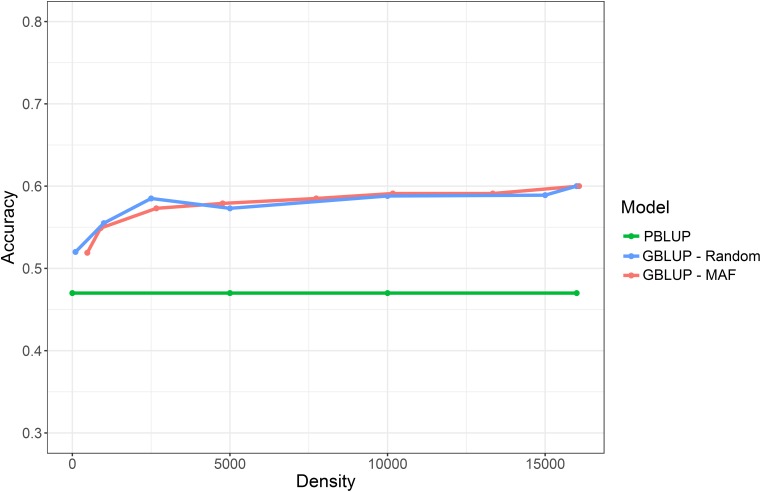
Prediction accuracy for the trait of SH when using PBLUP compared to GLUP using a range of different marker densities.

To evaluate the effect of marker density on genomic prediction accuracy, two strategies of obtaining lower density SNP panels were applied. The first used progressive increase of minor allele frequency (MAF) threshold, resulting in progressive decrease in SNP number (as described in Robledo et al. (2018). The second involved choosing subsets of SNPs for the low density panels at random. Using both approaches, significant reduction in SNP density had little impact on prediction accuracy until marker densities dropped below ∼2,500 SNPs. With the MAF approach, the genomic prediction accuracies obtained using the lower density SNP panels ranged from 0.59 (MAF >0.05; 13,337 SNPs) to 0.52 (MAF > 0.475; 474 SNPs) (**Table 2** and **Figure 2**). Using the random subsets the prediction accuracies ranged from 0.59 (15,000 SNPs) to 0.52 (100 SNPs). In all cases, the genomic prediction significantly outperformed pedigree prediction, even for low SNP densities, which has positive implications for future use of low-cost low-density SNP panels for genomic selection. The oyster genome is of a moderate size ∼0.6 gb (Zhang et al., 2012) and that could be related to the low number of markers needed to high prediction accuracies. Additionally, it is important to note that since this a relatively small sample set that contains a relatively limited number of families of known structure, a high level of relatedness between the training and validation sets is expected. A total of 23 nuclear families were included in the study, for which 15 dams and 16 sires were effective breeders, and resulted in a mix of full sibling and half sibling families (see **Supplementary File S1**). This may result in high levels of linkage disequilibrium across large chromosome segments, which could influence the genomic prediction accuracy estimates at low SNP densities. Nonetheless, this may be representative of typical aquaculture breeding schemes that utilize large full-sib families for “sib testing,” and where genomic prediction estimates with low density markers can give high prediction accuracy (Lillehammer et al., 2013; Tsai et al., 2015). Current limitations of the use of genomic selection in aquaculture companies relate to the cost of phenotype recording and genotyping, and the latter could be eased by the use of low cost genotyping which is directly related to number of SNPs to be typed. Our results suggest that low density SNP panels, as few as several hundred SNPs, may be sufficient to achieve the asymptote of prediction accuracy in a mixed family population of oysters that may be typical of an oyster breeding population.

**Table 2:** Genomic prediction values obtained for SH using decreasing marker densities.

Method	Approach	SNP N	Accuracy	Approach	SNP N	Accuracy
PBLUP	Pedigree	–	0.47			
GBLUP	MAF 0.01	16,076	0.6	Random	16,076	0.6
GBLUP	MAF 0.05	13,337	0.59	Random	15,000	0.59
GBLUP	MAF 0.1	10,167	0.59	Random	10,000	0.59
GBLUP	MAF 0.15	7,738	0.59	Random	5,000	0.57
GBLUP	MAF 0.25	4,768	0.58	Random	2,500	0.59
GBLUP	MAF 0.35	2,664	0.57	Random	1,000	0.56
GBLUP	MAF 0.45	898	0.55	Random	100	0.52
GBLUP	MAF 0.475	474	0.52			

## Conclusion

A recently developed medium density SNP array was used to evaluate the efficacy of different strategies for genomic prediction in a population of oysters derived from a commercial hatchery. Three growth-related traits were analyzed as exemplar polygenic traits (which was confirmed by GWAS). The three traits were found to be moderately heritable and showed high genetic correlation. Prediction accuracy for all traits was substantially higher using genomic prediction than pedigree-based prediction. Reduction in SNP marker density had little impact on prediction accuracy when the lower density SNP panels were chosen at random, implying only a fraction of the SNPs are required to obtain a marked increase in accuracy relative to pedigree-based prediction. These results suggest that the use of cost-effective genomic selection in oyster breeding could bring major benefits for the selection of polygenic traits, and may have commercial value for traits which are difficult to measure e.g., disease resistance.

## Author Contributions

AG and RH conceived the study. TB and RH designed the experimental structure. TB established and performed the experimental challenge. AG performed DNA extractions, genotype processing, and parentage assignment. AG and OM performed the quantitative genetic analyses. All authors contributed to drafting the manuscript.

## Conflict of Interest Statement

The authors declare that the research was conducted in the absence of any commercial or financial relationships that could be construed as a potential conflict of interest.

## Footnotes

1 http://reedmariculture.com/
