# Supplementary material for: Genomic Selection for Growth Traits in Pacific Oyster (Crassostrea gigas): Potential of Low-Density Marker Panels for Breeding Value Prediction
Source: Front Genet. 2018 Sep 19;9:391. doi: 10.3389/fgene.2018.00391 (PMC6156352; doi:10.3389/fgene.2018.00391)
Supplement: Supplementary file 1 [file Image_1.pdf]

Figure S1. Manhattan plots showing the GWAS results for A) Shell height B) Shell length and C) Wet weight.

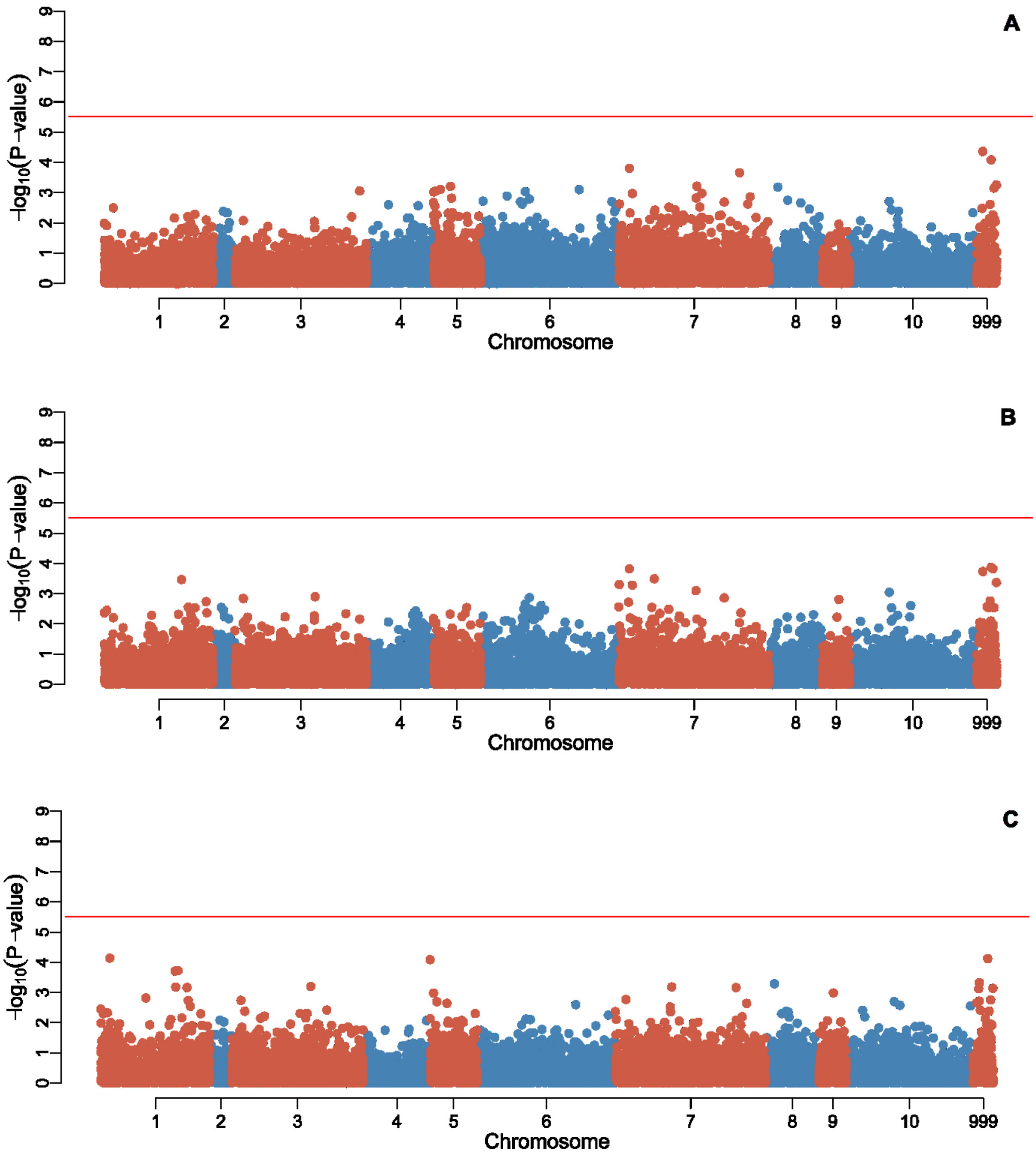

Markers sorted according to the 10 C. gigas linkage groups (LG) described in Gutierrez et al. (2018). LG shown as “999” correspond to markers not assigned to any LG.
