# Supplementary material for: Genomic Selection for Growth Traits in Pacific Oyster (Crassostrea gigas): Potential of Low-Density Marker Panels for Breeding Value Prediction
Source: Front Genet. 2018 Sep 19;9:391. doi: 10.3389/fgene.2018.00391 (PMC6156352; doi:10.3389/fgene.2018.00391)
Supplement: Supplementary file 2 [file Image_2.pdf]

Figure S2. Manhattan plots showing the GWAS results from BLUPF90 using a 10SNP window for A) Shell height B) Shell length and C) Wet weight.

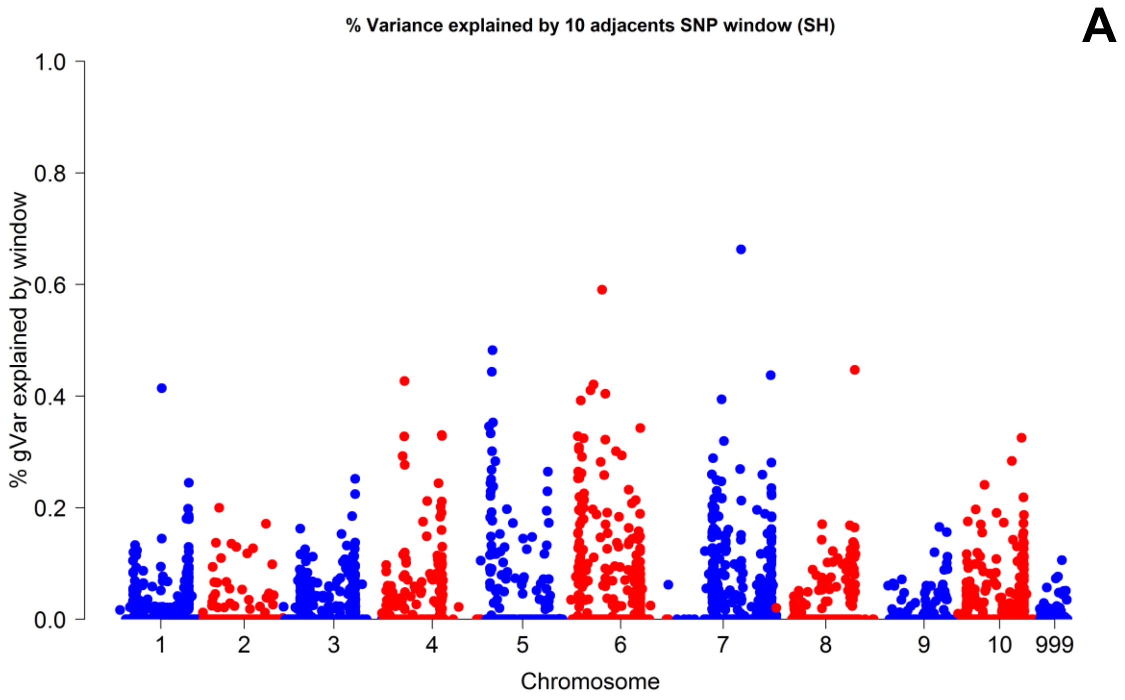

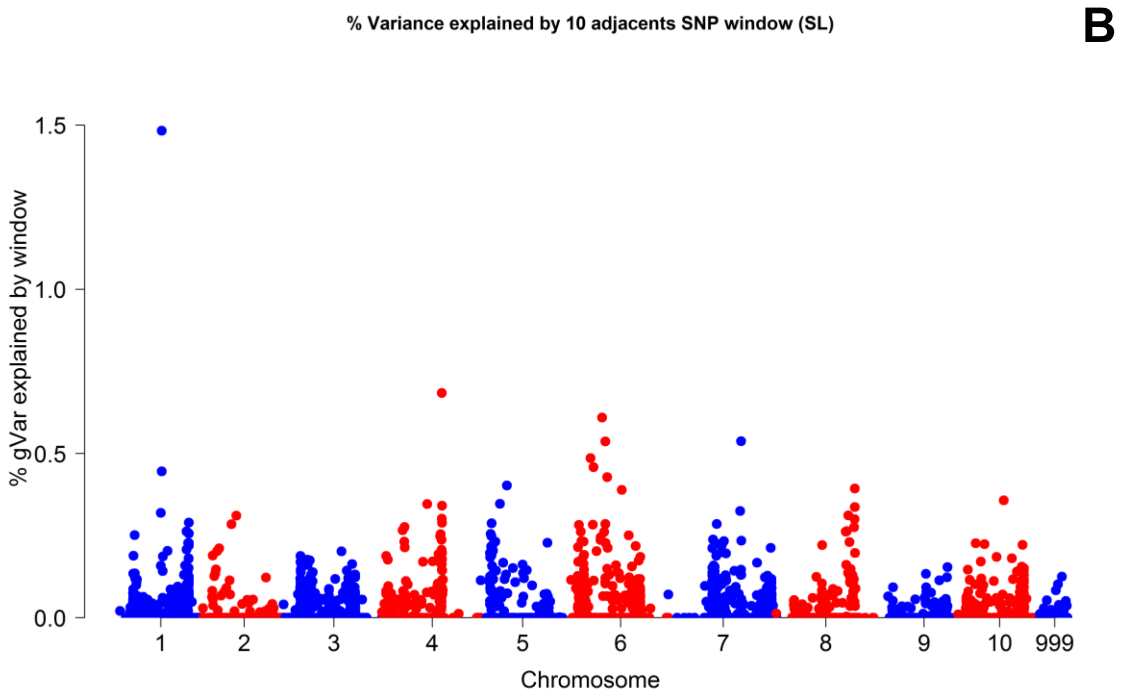

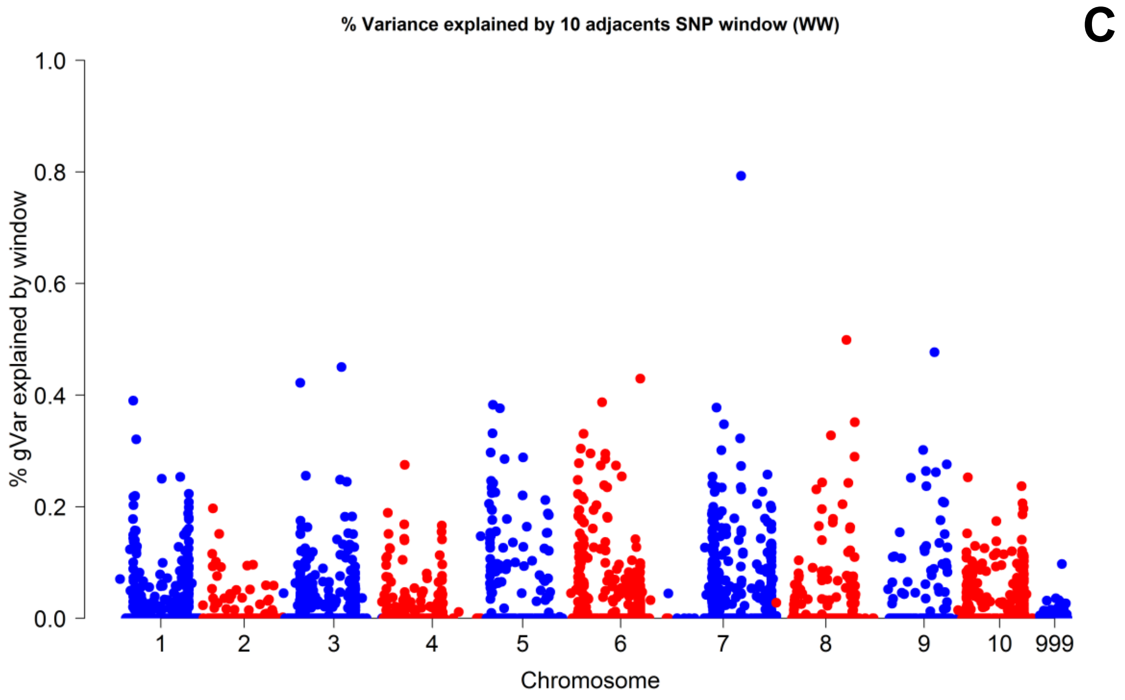

Markers sorted according to the 10 C. gigas linkage groups (LG) described in Gutierrez et al. (2018). LG shown as “999” correspond to markers not assigned to any LG.
