# Supplementary material for: Genomic Selection for Growth Traits in Pacific Oyster (Crassostrea gigas): Potential of Low-Density Marker Panels for Breeding Value Prediction
Source: Front Genet. 2018 Sep 19;9:391. doi: 10.3389/fgene.2018.00391 (PMC6156352; doi:10.3389/fgene.2018.00391)
Supplement: Supplementary file 3 [file Table_1.docx]

| SH |  |  |  |  |  |  |
| --- | --- | --- | --- | --- | --- | --- |
| Marker | LG | Position (cM) | p | q | p-value | PVE |
| AX-169173484 | NA | ** LG 4 (20.01) | 0.89 | 0.11 | 4.30E-05 | 0.022 |
| AX-169196669 | NA | ** LG 9 (73.01) | 0.92 | 0.08 | 8.20E-05 | 0.021 |
| AX-169176391 | 7 | 36.21 | 0.9 | 0.1 | 1.60E-04 | 0.02 |
| AX-169156708 | 7 | 63.04 | 0.91 | 0.09 | 2.20E-04 | 0.018 |
| AX-169208860 | NA | ** LG 8 (54.37) | 0.9 | 0.1 | 5.60E-04 | 0.016 |
| AX-169178317 | 7 | 54.39 | 0.73 | 0.27 | 6.10E-04 | 0.015 |
|  |  |  |  |  |  |  |
| SL |  |  |  |  |  |  |
| Marker | LG | position (cM) | p | q | p-value | PVE |
| AX-169196669 | NA | ** LG 9 (73.01) | 0.92 | 0.08 | 1.40E-04 | 0.021 |
| AX-169200900 | NA | ** LG 7 (49.24) | 0.95 | 0.05 | 1.50E-04 | 0.021 |
| AX-169176391 | 7 | 36.21 | 0.9 | 0.1 | 1.50E-04 | 0.021 |
| AX-169173484 | NA | ** LG 4 (20.01) | 0.89 | 0.11 | 1.90E-04 | 0.019 |
| AX-169180741 | 7 | 43.23 | 0.79 | 0.21 | 3.20E-04 | 0.017 |
| AX-169182508 | 1 | 53.55 | 0.95 | 0.05 | 3.40E-04 | 0.017 |
|  |  |  |  |  |  |  |
| WW |  |  |  |  |  |  |
| Marker | LG | position (cM) | p | q | p-value | PVE |
| AX-169165496 | 1 | 27.94 | 0.81 | 0.19 | 7.30E-05 | 0.021 |
| AX-169196669 | NA | ** LG 9 (73.01) | 0.92 | 0.08 | 7.60E-05 | 0.021 |
| AX-165319118 | 5 | 25.77 | 0.86 | 0.14 | 8.20E-05 | 0.022 |
| AX-169182508 | 1 | 53.55 | 0.95 | 0.05 | 1.90E-04 | 0.018 |
| AX-169204106 | 1 | 53.3 | 0.85 | 0.15 | 1.90E-04 | 0.018 |
| AX-169173484 | NA | ** LG 4 (20.01) | 0.89 | 0.11 | 4.70E-04 | 0.016 |
| LG, linkage group. **Position of the nearest marker in the linkage map, shown as LG (cM) | | | | | | |

Table S1. GenABEL GWAS results for the analysis of shell height (SH), shell length (SL) and wet weight (WW).
