# Supplementary material for: Genomic Selection for Growth Traits in Pacific Oyster (Crassostrea gigas): Potential of Low-Density Marker Panels for Breeding Value Prediction
Source: Front Genet. 2018 Sep 19;9:391. doi: 10.3389/fgene.2018.00391 (PMC6156352; doi:10.3389/fgene.2018.00391)
Supplement: Supplementary file 4 [file Table_2.docx]

| SH |  |  |  |  |
| --- | --- | --- | --- | --- |
| Marker | LG | Window start (cM) | Window end (cM) | gVar(%) |
| AX-169175178 | 7 | 73.23 | 73.23 | 0.66 |
| AX-169158130 | 6 | 39.94 | 40.82 | 0.59 |
| AX-169189790 | 5 | 11.94 | 12.19 | 0.48 |
| AX-169166143 | 8 | 78.99 | 78.99 | 0.45 |
| AX-169180307 | 5 | 11.19 | 11.31 | 0.44 |
| AX-169192329 | 7 | 103.03 | 103.03 | 0.44 |
| AX-169165874 | 4 | 22.56 | 22.69 | 0.43 |
| AX-169159251 | 6 | 31.36 | 31.36 | 0.42 |
| AX-169162130 | 1 | 48.61 | 48.61 | 0.41 |
| AX-169156847 | 6 | 28.21 | 30.1 | 0.41 |
|  |  |  |  |  |
| SL |  |  |  |  |
| Marker | LG | Window start (cM) | Window end (cM) | gVar(%) |
| AX-169162130 | 1 | 48.61 | 48.61 | 1.48 |
| AX-169208403 | 4 | 60.04 | 60.3 | 0.68 |
| AX-169158130 | 6 | 39.94 | 40.82 | 0.61 |
| AX-169175178 | 7 | 73.23 | 73.23 | 0.54 |
| AX-169183085 | 6 | 43.2 | 43.2 | 0.54 |
| AX-169156847 | 6 | 28.21 | 30.1 | 0.49 |
| AX-169159251 | 6 | 31.36 | 31.36 | 0.46 |
| AX-169190876 | 1 | 48.61 | 48.73 | 0.45 |
| AX-169180941 | 6 | 45.08 | 45.08 | 0.43 |
| AX-169181319 | 5 | 26.24 | 26.87 | 0.40 |
|  |  |  |  |  |
| WW |  |  |  |  |
| Marker^a^ | LG | Window start (cM)^b^ | Window end (cM)^c^ | gVar(%)^d^ |
| AX-169175178 | 7 | 73.23 | 73.23 | 0.79 |
| AX-169186907 | 8 | 70.6 | 70.72 | 0.50 |
| AX-169176380 | 9 | 61.34 | 62.72 | 0.48 |
| AX-169158926 | 3 | 58.03 | 59.55 | 0.45 |
| AX-169161990 | 6 | 78.25 | 78.25 | 0.43 |
| AX-169189280 | 3 | 16.73 | 16.73 | 0.42 |
| AX-169169076 | 1 | 19.81 | 19.81 | 0.39 |
| AX-169158130 | 6 | 39.94 | 40.82 | 0.39 |
| AX-169178065 | 5 | 12.44 | 12.56 | 0.38 |
| AX-169159303 | 7 | 48.58 | 48.71 | 0.38 |

Table S2. BLUPF90 GWAS results for the analysis of shell height (SH), shell length (SL) and wet weight (WW), using a 10 SNP window approach.

^a^ Marker shown is the first in the 10 SNP window. ^b^ Start position on the linkage map for the starting SNP; ^c^ Position of the 10^th^ SNP included in the window. ^d^ gVar% represents the percentage of genetic variance explained by the SNP.
